# Supplementary figures and images for: Diversity of Aβ aggregates produced in a gut-based Drosophila model of Alzheimer’s disease
Source: PLoS One. 2025 Jul 8;20(7):e0314832. doi: 10.1371/journal.pone.0314832 (PMC12237042; doi:10.1371/journal.pone.0314832)

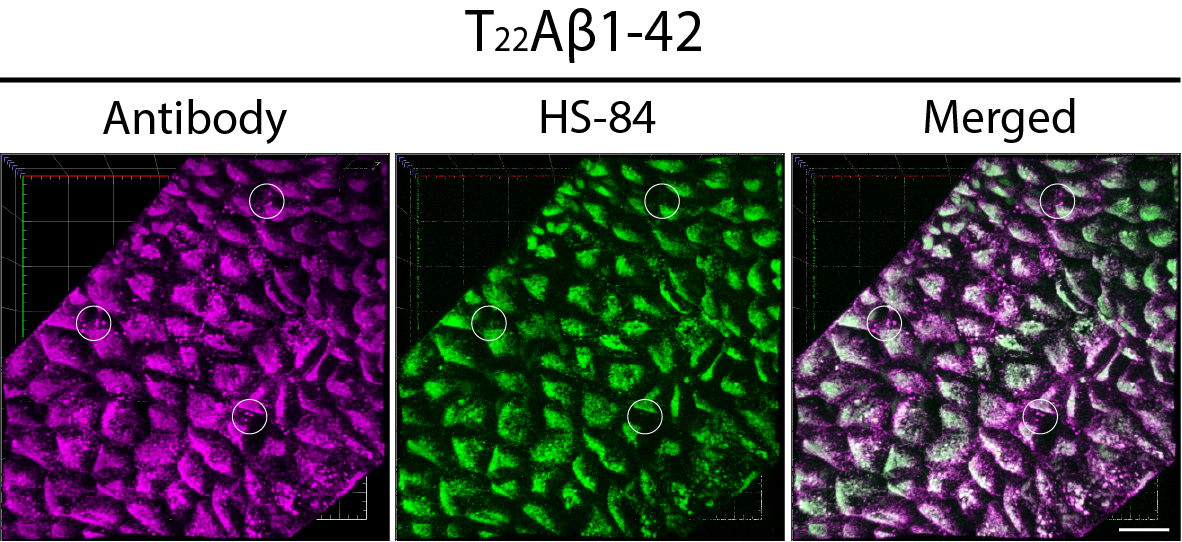

Supplement: S1 Fig — Detection of Aβ species in the anterior midgut of Drosophila flies expressing the tandem construct T22Aβ1–42 in the enterocytes using the Myo31DF driver. The confocal microscope three-dimensional (3D) images show gut tissue costained with Mabtech anti-human Aβ antibody (magenta) and LCO ligand HS-84 (green) 8 days post eclosion. White circles: Aβ species labeled by the antibody and not HS-84. Scale bar, 40 μm. (PNG) [file pone.0314832.s001.png]

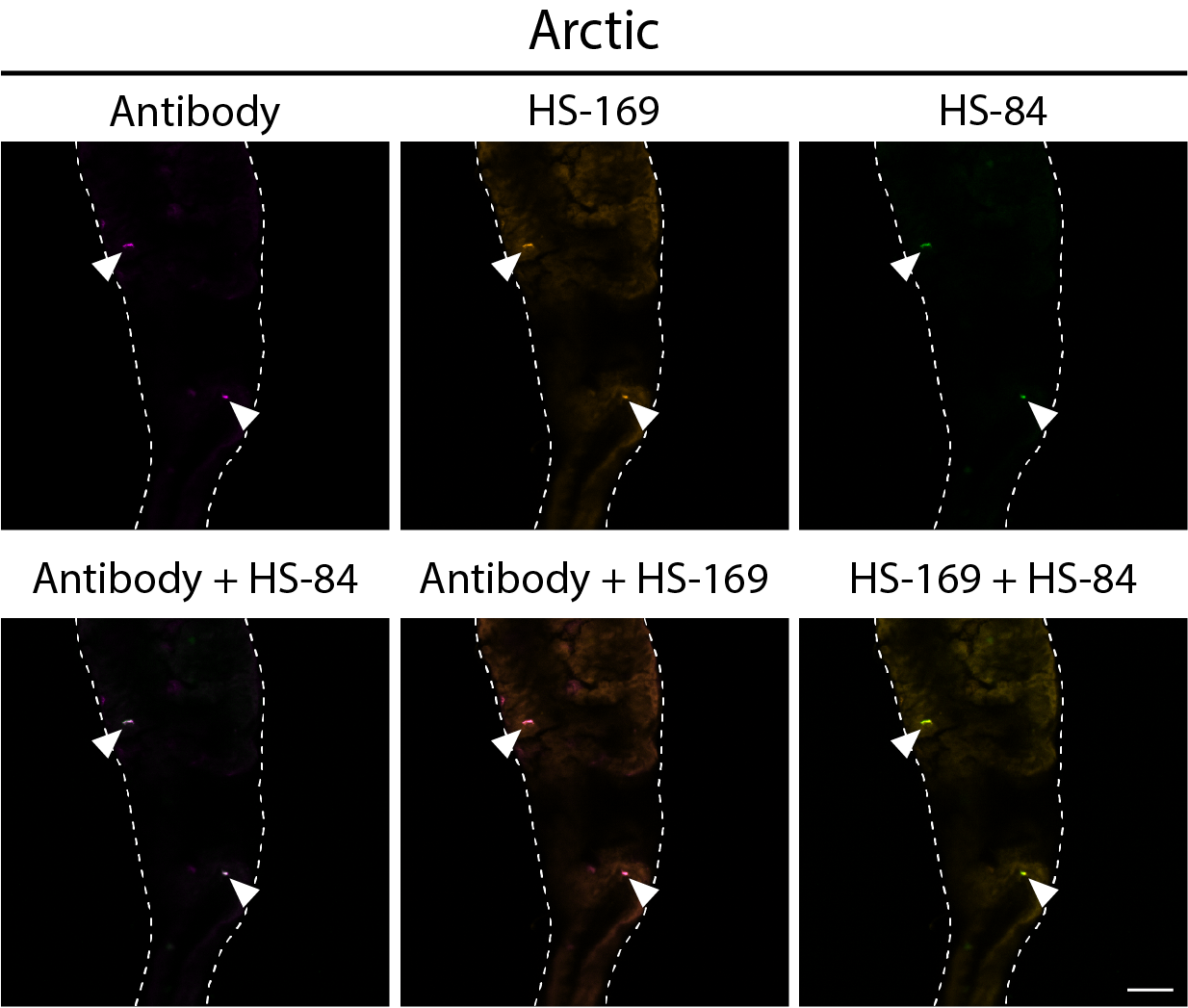

Supplement: S2 Fig — Detection of Aβ species in the anterior midgut of Drosophila flies expressing the Arctic mutant of Aβ1–42 in the enterocytes using the Myo31DF driver. The confocal microscope single-plane images show gut tissue costained with Mabtech anti-human Aβ antibody (magenta) and LCO ligands HS-84 (green) and HS-169 (orange) 8 days post eclosion. White arrowheads: Aβ species labeled by all three staining agents. Scale bar, 50 μm. (PNG) [file pone.0314832.s002.png]
