# Supplementary material for: Diversity of Aβ aggregates produced in a gut-based Drosophila model of Alzheimer’s disease
Source: PLoS One. 2025 Jul 8;20(7):e0314832. doi: 10.1371/journal.pone.0314832 (PMC12237042; doi:10.1371/journal.pone.0314832)
Supplement: Attachment — Submitted filename: Author response to reviewers.docx [file pone.0314832.s004.docx]

# From Editor

# As presented in the appended comments below both reviewers express significant questions regarding technical aspects of the model itself questioning its validity. These concerns must be thoroughly addressed, with additional experiments as necessary. Clearly additional experiments are necessary to assess more than a single point aggregation assessment.

# Both reviewers raise concerns regarding the use of lifespan as the single in vivo toxicity indicator and this concern must be thoroughly addressed.

# Moreover, a number of additional concerns are raised regarding data presentation, statistical analyses and interpretation by both reviewers and these have to also be addressed point by point.

# I recognise that these required changes including additional experiments will take time beyond the automatically allotted, so fell free to ask for additional time to complete these.

# Please submit your revised manuscript by Feb 16 2025 11:59PM. If you will need more time than this to complete your revisions, please reply to this message or contact the journal office at [plosone@plos.org](mailto:plosone@plos.org). When you're ready to submit your revision, log on to https://www.editorialmanager.com/pone/ and select the 'Submissions Needing Revision' folder to locate your manuscript file. Please include the following items when submitting your revised manuscript:

# A rebuttal letter that responds to each point raised by the academic editor and reviewer(s). You should upload this letter as a separate file labeled 'Response to Reviewers'.

# A marked-up copy of your manuscript that highlights changes made to the original version. You should upload this as a separate file labeled 'Revised Manuscript with Track Changes'.

# An unmarked version of your revised paper without tracked changes. You should upload this as a separate file labeled 'Manuscript'

**Reviewers**

Manuskript: Designing a Novel Drosophila Model of Alzheimer’s Disease to Study Aβ Proteotoxicity in the Digestive Tract.

Reviewer's Responses to Questions

**Comments to the Author**

1. Is the manuscript technically sound, and do the data support the conclusions?

The manuscript must describe a technically sound piece of scientific research with data that supports the conclusions. Experiments must have been conducted rigorously, with appropriate controls, replication, and sample sizes. The conclusions must be drawn appropriately based on the data presented.

Reviewer #1: No

Reviewer #2: No

2. Has the statistical analysis been performed appropriately and rigorously?

Reviewer #1: No

Reviewer #2: No

3. Have the authors made all data underlying the findings in their manuscript fully available?

The [PLOS Data policy](https://eur01.safelinks.protection.outlook.com/?url=http%3A%2F%2Ftrack.editorialmanager.com%2FCL0%2Fhttp%3A%252F%252Fwww.plosone.org%252Fstatic%252Fpolicies.action%2523sharing%2F1%2F010f019428ffafe4-221f44f1-931d-443c-8905-fcb9899bc7f9-000000%2FmscbvR1p2NdnK6nUbamADVMgHGDlbvvmkNxMIHKKLaY%3D191&data=05%7C02%7Cann-christin.brorsson%40liu.se%7Caf520a37701d4b04b0d108dd2ff2f55f%7C913f18ec7f264c5fa816784fe9a58edd%7C0%7C0%7C638719442725015915%7CUnknown%7CTWFpbGZsb3d8eyJFbXB0eU1hcGkiOnRydWUsIlYiOiIwLjAuMDAwMCIsIlAiOiJXaW4zMiIsIkFOIjoiTWFpbCIsIldUIjoyfQ%3D%3D%7C0%7C%7C%7C&sdata=Dmi1mi7c7uG6zzMN5plqmgsYNBT1C0m7A67J6sjAOgM%3D&reserved=0) requires authors to make all data underlying the findings described in their manuscript fully available without restriction, with rare exception (please refer to the Data Availability Statement in the manuscript PDF file). The data should be provided as part of the manuscript or its supporting information, or deposited to a public repository. For example, in addition to summary statistics, the data points behind means, medians and variance measures should be available. If there are restrictions on publicly sharing data—e.g. participant privacy or use of data from a third party—those must be specified.

Reviewer #1: No

Reviewer #2: Yes

4. Is the manuscript presented in an intelligible fashion and written in standard English?

PLOS ONE does not copyedit accepted manuscripts, so the language in submitted articles must be clear, correct, and unambiguous. Any typographical or grammatical errors should be corrected at revision, so please note any specific errors here.

Reviewer #1: Yes

Reviewer #2: Yes

5. Review Comments to the Author

**Reviewer #1:** This paper describes the characterisation of the aggregation state of two previously published Aß constructs, a wt double Aß and an arctic Aß. They describe different aggregation states for the two constructs and attempt to correlate this to their toxicity. The authors do all this in fly guts. Although fly guts are more accessible than neurons, and could potentially be used for biochemical characterisation of Aß aggregation, the authors should explain in more detail why this is a relevant or useful model. There are also some issues that need addressing before the manuscript is ready for publication.

1. It is not totally clear what model is used, do these Aß models contain a secretion model? Is it the same for the two? Are both peptides cleaved and secreted in this model or are the Aß peptides measured here intracellular?

**Author response:** Yes – the expressed Aβ peptide or construct has a secretory signal peptide. This information has been added in the Materials and methods section of the paper.

2. Quantifications for the stainings are missing: HS-84 and HS-169 signals should be normalised and quantified with replicates. Co-localisation with Aß signal should also be quantified.

**Author response:** We agree – quantifications of the co-staining have been performed and added to the paper, see Figure 4B.

3. The authors claimed that they observed similar toxic effect (based on similar median survival days) and calculated the extent of reduction in lifespan per unit of Aβ. This was based on the assumption that the reduction in lifespan (i.e. toxicity) and Abeta levels **are always linearly correlated without saturation effect**. However, it is possible that Abeta levels (of a certain form or different forms combined) need to fall in a range to be correlated to the toxic outcomes, and further increasing its levels will not become more toxic? Using geneswitch systems at a gradient of RU doses may help determine whether the toxicity from Abeta is already saturated.

**Author response:** Interesting idea about saturation effect. However, we do not prefer to use the geneswitch systems since recent research has found adverse effects triggered by activation of the GeneSwitch system (see Zappia (2024)). In addition, we believe that the fourfold higher aggregation load (from the MSD analysis) and the 40-fold higher aggregation load (from the quantifications of the histology data) found in the T22Aβ1-42 flies compared to the Arctic flies at day 8, when the flies are starting to die, clearly shows that more aggregates accumulate in the T22Aβ1-42 flies compared to the Arctic flies just before they start to die. This result reveals that the flies can cope with a higher load of T22Aβ1-42 aggregates compared to Arctic aggregates before they start to die.

Zappia, M. P. (2024), et.al, The RU486-dependent activation of the GeneSwitch system in adult muscles leads to severe adverse effects in Drosophila. G3 (Bethesda, Md.), 14(5), jkae039. https://doi.org/10.1093/g3journal/jkae039

4. The authors base their conclusions on a single point for Aß aggregation. It is possible that the Arctic mutation leads to earlier aggregation, relative to the wt Aß constructs. The quantification of soluble/insoluble Aß should be performed at different timepoints, to show that they increase with similar dynamics.

**Author response:** We agree that this would be an interesting experiment to find out if the Arctic mutation leads to earlier aggregation, relative to the wt Aß constructs. However, in this study, we aimed to investigate the accumulation and distribution of aggregates as close as possible to the onset of toxic effects in the flies. This was chosen to be on day 8, the time point at which the flies are just beginning to die.

4. The authors used lifespan as the only readout of Aβ toxicity. Do these gut models of Aβ have other phenotypic readouts, for example gut leakage?

**Author response:** To deepen our understanding of the toxicity, an analysis of gut leakage has been performed and added to the paper. This experiment was done by feeding the flies the blue dye erioglaucine disodium salt and then studying the distribution of the stain in the fly hemocoel. Spreading of the blue dye in the hemocoel and body indicates gut leakage, which is a sign of toxicity. This result has been added to the manuscript (see figure 2).

5. The authors chose day 8 flies because “day 8 was near the median survival times for both genotypes”, but is Day8 really close to median survival (shown in Fig1A)?

**Author response:** Day 8 was chosen because it is the time point when the flies are just starting to die, but there are still enough flies alive to collect the necessary number for the experiment. Consequently, the histological and MSD analyses could not be performed at a later time point. This has been clarified in the manuscript where the following text have been added “*Day 8 is fairly close to the median survival time for both genotypes (40), marking the point when the flies begin to die. However, there are still enough flies alive to gather the required number for the experiment”*.

6. Why is there so much more Aβ in the T22Aβ1-42 line than that in the Arctic line? Is it due to different transcription levels? Or degradation mechanisms clearing them at different rates? A qPCR showing transcription levels should be included to show whether transcription was equivalent.

**Author response:** In this study, our main goal was to examine the aggregation load in T22Aβ1-42 flies and Arctic flies and to investigate the diversity of these Aβ aggregates. Although the different aggregates may be cleared to varying extents, the toxicity is very similar between these two Aβ fly genotypes. Therefore, our primary interest was to explore the differences in the accumulation and distribution of aggregates in these two fly systems at the onset of the toxic effect, as measured by the survival assay. Consequently, we do not believe it is necessary to run qPCR, as our interest is in the level of aggregates and their relationship to toxicity.

7. Statistical analysis in Fig 4 or 5 is missing. This should be presented.

**Author response:** Yes – statistical analysis has been added to these figures (now named figure 5 and 6).

8. Expressing Aβ in neurons would be more relevant to disease context. Why did the authors focus on the gut? Authors should comment in more detail as to **the usefulness of this model**.

**Author response:** The aim of this study was to investigate the diversity of Aβ aggregates produced by flies expressing the dimeric construct of Aβ1-42 and flies expressing the Aβ peptide with the Arctic mutation E22G. Our previous study revealed that expression of Arctic and T22Aβ1-42 in the fly gut resulted in different Aβ loads despite a similar survival rate (see Elovsson et al. (2024), *A Novel Drosophila Model of Alzheimer’s Disease to Study Aβ Proteotoxicity in the Digestive Tract*, IJMS 25(4), 2105). Therefore, we decided to continue our research using this model, where Aβ is expressed in the enterocytes, to explore these differences in Aβ load more thoroughly. This model is very useful for studying the properties of Aβ aggregates because the accumulation of aggregates is quite high (especially for T22Aβ1-42), which facilitates studies of these aggregates.

**Reviewer #2:** This study investigated the heterogeneity of Aβ aggregates produced in the digestive tract in fruit flies expressing the Aβ1-42 peptide with the Arctic mutation E22G (Arctic fruit flies) or a dimeric construct of Aβ1-42 (T22Aβ1-42 fruit flies). Although fruit flies expressing these two Tg Aβ strains exhibited a similar degree of lifespan reduction, the Aβ produced by these flies showed different LCO staining patterns and differed in their solubility in Gua-HCl. Based on these results, the authors claim to have demonstrated that different Aβ aggregates have different pathological effects. It is interesting to note that the two fruit fly models they used had similar levels of toxicity despite different degrees of expression and aggregation. However, it is well known that the Arctic mutation is associated with increased aggregation and increased toxicity compared to wild-type Aβ. The experimental evidence presented in this study is unreliable and preliminary and does not sufficiently support their claims. Furthermore, some of the results appear to be a repetition or reuse of the authors' previous work. Here are the main points of criticism.

1) Alzheimer's disease is a neurodegenerative disease. The validity of the model of Aβ expression in the gut is questionable. It remains to be seen if the observations of this study are replicated in the brain.

**Author response:** The aim of this study was to investigate the diversity of Aβ aggregates produced by flies expressing the dimeric construct of Aβ1-42 and flies expressing the Aβ peptide with the Arctic mutation E22G. Our previous study revealed that expression of Arctic and T22Aβ1-42 in the fly gut resulted in different Aβ loads despite a similar survival rate (see Elovsson et al. (2024), *A Novel Drosophila Model of Alzheimer’s Disease to Study Aβ Proteotoxicity in the Digestive Tract*, IJMS 25(4), 2105). Therefore, we decided to continue our research using this model, where Aβ is expressed in the enterocytes, to explore these differences in Aβ load more thoroughly. This model is very useful for studying the properties of Aβ aggregates because the accumulation of aggregates is quite high (especially for T22Aβ1-42), which facilitates studies of these aggregates.

2) Figure 1A appears to be a figure already used by the authors in a previously published paper (a).

a. Elovsson G, Klingstedt T, Brown M, Nilsson KPR, Brorsson AC. A Novel Drosophila Model of Alzheimer's Disease to Study Aβ Proteotoxicity in the Digestive Tract. Int J Mol Sci. 2024 Feb 9;25(4):2105. doi: 10.3390/ijms25042105.

**Author response:** In the paper we have clearly stated that Figure 1A is an illustration of a survival study from a previous paper. It is therefore appropriate to use this figure in this paper.

3) Figure 1B, C, Figure 2, 3, etc. should be measured by quantitative methods. The single figure presented is insufficient to be reliable data. Comparison of more objects and more locations is needed.

**Author response:** Quantifications of the co-staining have been performed and added to the paper, see Figure 4B.

4) In Figure 4, the amount of Aβ accumulated in T22Aβ1-42 fruit flies appears to be about 4 times greater than that in Arctic flies, but the differences in Figures 2 and 3 appear to be much greater.

**Author response:** Yes, we agree that the differences you mention above appear to be greater in the histology results compared to the MSD results. This could be due to the use of different antibodies in the experiments or the possibility that not all T22Aβ1-42 aggregates are dissolved in ≤5 M Gua-HCl in the MSD experiment, leaving some aggregates in the pellet after the final extraction step. Nevertheless, both experiments show that more aggregates accumulate in the T22Aβ1-42 flies compared to the Arctic flies.

5) Only one indicator of toxicity was presented, lifespan. However, lifespan is not a direct indicator of Aβ toxicity. Other parameters such as cell death should be used.

**Author response:** An additional toxic parameter has been added to the paper, which includes an analysis of gut leakage. This experiment was done by feeding the flies the blue dye erioglaucine disodium salt and then studying the distribution of the stain in the fly hemocoel. Spreading of the blue dye in the hemocoel and body indicates gut leakage, which is a sign of toxicity. This result has been added to the manuscript (see figure 2).

6) Lack of appropriate controls in Figures 2 and 3. It is questionable whether the two dyes used stained at the same brightness. The staining of the two dyes may be different depending on various factors such as staining method, confocal laser intensity, and post-experiment observation time. In Figure 2, we also observe green areas that stain with HS-84 but not with the antibody. How to explain this is questionable.

**Author response:**

Since these images represent a co-staining, the samples have been treated equally. When it comes to confocal microscopy, it is not possible to use exactly the same brightness/gain since the ligands have different fluorescent properties. Thus, we have ensured that the brightness of one of the co-stained aggregate with high intensity is adjusted to display an equally strong signal for all three agents. For the T22Aβ1-42 flies, this aggregate is pointed out by the bottom left arrow in figure 2 (now Figure 3).

To ensure proper analysis of HS-84 and HS-169 signals from the binding of the ligands to Aβ aggregates, co-staining of these two ligands was performed using an antibody against Aβ. Only the signals from the ligands that co-localized with the antibody were analyzed. Quantifications of the co-staining have been performed and added to the paper (see Figure 4B).
