# Supplementary material for: Diversity of Aβ aggregates produced in a gut-based Drosophila model of Alzheimer’s disease
Source: PLoS One. 2025 Jul 8;20(7):e0314832. doi: 10.1371/journal.pone.0314832 (PMC12237042; doi:10.1371/journal.pone.0314832)
Supplement: Attachment — Submitted filename: Author response to reviewers 250613.docx [file pone.0314832.s005.docx]

# From Editor

# Dear Dr. Brorsson, Thank you for submitting your manuscript to PLOS ONE. After careful consideration, we feel that it has merit but does not fully meet PLOS ONE’s publication criteria as it currently stands. Therefore, we invite you to submit a revised version of the manuscript that addresses the points raised during the review process. Please include the following items when submitting your revised manuscript:

# A rebuttal letter that responds to each point raised by the academic editor and reviewer(s). You should upload this letter as a separate file labeled 'Response to Reviewers'.

# A marked-up copy of your manuscript that highlights changes made to the original version. You should upload this as a separate file labeled 'Revised Manuscript with Track Changes'.

# An unmarked version of your revised paper without tracked changes. You should upload this as a separate file labeled 'Manuscript'.

**Reviewer's Responses to Questions**

Comments to the Author

1. If the authors have adequately addressed your comments raised in a previous round of review and you feel that this manuscript is now acceptable for publication, you may indicate that here to bypass the “Comments to the Author” section, enter your conflict of interest statement in the “Confidential to Editor” section, and submit your "Accept" recommendation.

Reviewer #1: (No Response)

Reviewer #2: (No Response)

2. Is the manuscript technically sound, and do the data support the conclusions?

The manuscript must describe a technically sound piece of scientific research with data that supports the conclusions. Experiments must have been conducted rigorously, with appropriate controls, replication, and sample sizes. The conclusions must be drawn appropriately based on the data presented.

Reviewer #1: Partly

Reviewer #2: No

3. Has the statistical analysis been performed appropriately and rigorously?

Reviewer #1: No

Reviewer #2: No

4. Have the authors made all data underlying the findings in their manuscript fully available?

The PLOS Data policy requires authors to make all data underlying the findings described in their manuscript fully available without restriction, with rare exception (please refer to the Data Availability Statement in the manuscript PDF file). The data should be provided as part of the manuscript or its supporting information, or deposited to a public repository. For example, in addition to summary statistics, the data points behind means, medians and variance measures should be available. If there are restrictions on publicly sharing data—e.g. participant privacy or use of data from a third party—those must be specified.

Reviewer #1: Yes

Reviewer #2: Yes

5. Is the manuscript presented in an intelligible fashion and written in standard English?

PLOS ONE does not copyedit accepted manuscripts, so the language in submitted articles must be clear, correct, and unambiguous. Any typographical or grammatical errors should be corrected at revision, so please note any specific errors here.

Reviewer #1: Yes

Reviewer #2: Yes

6. Review Comments to the Author

Please use the space provided to explain your answers to the questions above. You may also include additional comments for the author, including concerns about dual publication, research ethics, or publication ethics. (Please upload your review as an attachment if it exceeds 20,000 characters)

**Reviewer #1:** The authors have addressed some of our concerns. A few points still need addressing:

-there is no Fig 4B in the manuscript that I could find

**Author response:** Yes – that is correct, there is only one Figure 4 that is referred to in the manuscript and that has been resubmitted.

-there is no statistical analysis for the guts phenotype

**Author response:** Statistical analysis of the gut phenotype yielded a P-value greater than 0.05, which strongly suggests that the result is likely under the null hypothesis. Although this intriguing phenotype was observed in Arctic flies 8 days post-eclosion, it is not statistically significant. Therefore, in the paper, we cautiously interpret this finding as a potential indication of a toxic mechanism in Arctic flies that differs from the toxic events in the T22Aβ1-42 flies.

-the gut phenotype is not discussed, if there is a significant difference, what does this mean for toxicity.

**Author response:** See the answer above

-the conclusions should be toned down, as the authors have not been able to show that the toxicity does not have a thresholding effect and anything higher than a certain amount of Aß will always give the same toxicity, they should add a sentence to this effect in the discussion

**Author response:** The discussion has been updated at line 378-384. *“Although the survival data revealed a similar reduction in median lifespan between the two fly genotypes, a potential saturation effect of Aβ accumulation should be considered which could mean that once Aβ levels reach a certain threshold, additional accumulation does not lead to increased toxicity. However, the outcome from the calculation of the proteotoxic effect showed that the Aβ1-42 pool produced in Arctic flies was 4 times more proteotoxic than the Aβ1-42 pool produced in T22Aβ1-42 flies revealing that the toxic effect per Aβ unit (pg/fly) is higher in the Arctic flies compared to the T22Aβ1-42 flies.”*

**Reviewer #2:** PONE-D-24-52919R1

Diversity of Aβ aggregates produced in a gut-based Drosophila model of Alzheimer’s disease

Elovsson et al.

There remain concerns regarding the novelty of this study. As pointed out in the previous review, it is well established that the Arctic mutation is associated with increased aggregation and enhanced toxicity compared to wild-type Aβ (Iijima et al., 2008; Perálvarez-Marín et al., 2009). Moreover, the exact same model expressing these Aβ in the gut has already been published by the current authors themselves (Elovsson et al., 2024). It is therefore unclear what additional advancement this manuscript offers beyond what has already been reported.

**Author response:** The novelty of this study lies in the detailed *in vivo* characterization of Aβ aggregates formed in Arctic flies and in flies expressing a dimeric construct of Aβ1–42. Specifically, we demonstrate how these aggregates can be analyzed for their morphological properties using luminescent conjugated oligothiophenes (LCOs), which selectively bind to Aβ aggregates. Furthermore, we reveal distinct differences in the *in vivo* aggregation behavior of the two Aβ variants: the T22Aβ1–42 peptide tends to form aggregates that are largely insoluble in 5 M guanidine hydrochloride (Gua), whereas the Arctic peptide forms aggregates that are more readily solubilized at lower Gua concentrations. These findings provide valuable insights into the aggregation dynamics of Aβ peptides and offer a foundation for future studies aimed at elucidating the relationship between Aβ aggregation and its toxic mechanisms, ultimately contributing to a better understanding of Alzheimer’s disease pathogenesis.

Perálvarez-Marín A, Mateos L, Zhang C, Singh S, Cedazo-Mínguez A, Visa N, Morozova-Roche L, Gräslund A, Barth A. Influence of residue 22 on the folding, aggregation profile, and toxicity of the Alzheimer's amyloid beta peptide. Biophys J. 2009 Jul 8;97(1):277-85.

Iijima K, Chiang HC, Hearn SA, Hakker I, Gatt A, Shenton C, Granger L, Leung A, Iijima-Ando K, Zhong Y. Abeta42 mutants with different aggregation profiles induce distinct pathologies in Drosophila. PLoS One. 2008 Feb 27;3(2):e1703. doi: 10.1371/journal.pone.0001703. PMID: 18301778; PMCID: PMC2250771.

Elovsson G, Klingstedt T, Brown M, Nilsson KPR, Brorsson AC. A Novel Drosophila Model of Alzheimer's Disease to Study Aβ Proteotoxicity in the Digestive Tract. Int J Mol Sci. 2024 Feb 9;25(4):2105. doi: 10.3390/ijms25042105. PMID: 38396782; PMCID: PMC10888607.

Minor point

Statistical error in Figure 6A: The result does not appear to be non-significant (ns) as indicated.

**Author response:** This result is not statistically significant, likely due to the extremely low levels of Aβ detected (below 1 pg per fly) which are near the lower limit of the assay’s sensitivity.

7. PLOS authors have the option to publish the peer review history of their article (what does this mean?). If published, this will include your full peer review and any attached files.

If you choose “no”, your identity will remain anonymous but your review may still be made public.

Do you want your identity to be public for this peer review? For information about this choice, including consent withdrawal, please see our Privacy Policy.

Reviewer #1: No

Reviewer #2: No
